# Neuropsychological Evaluations in Limbic Encephalitis

**DOI:** 10.3390/brainsci11050576

**Published:** 2021-04-29

**Authors:** Juri-Alexander Witt, Christoph Helmstaedter

**Affiliations:** Department of Epileptology, University Hospital Bonn (UKB), 53127 Bonn, Germany; christoph.helmstaedter@ukbonn.de

**Keywords:** limbic encephalitis, autoimmune epilepsy, neuropsychology, cognition, behavior, monitoring, assessment, diagnostics, memory, auto-antibodies

## Abstract

Limbic encephalitis (LE) can cause dynamic and permanent impairment of cognition and behavior. In clinical practice, the question arises as to which cognitive and behavioral domains are affected by LE and which assessment is suited to monitor the disease progress and the success of treatment. Current findings on cognition and behavior in LE are reviewed and discussed based on current guidelines and consensus papers. In addition, we outline approaches for the neuropsychological monitoring of LE and its treatment. Dependent on disease acuity and severity, LE leads to episodic long-term memory dysfunction in different variants (e.g., anterograde memory impairment, accelerated long-term forgetting, and affection of autobiographical memory) and executive deficits. In addition, affective disorders are very common. More severe psychiatric symptoms may occur as well. In the course of the disease, dynamic phases with functional recovery must be differentiated from residual defect states. Evidence-based neuropsychological diagnostics should be conducted ideally before treatment initiation and reassessments are indicated when any progress is suggested, and when decisive anti-seizure or immunomodulatory treatment changes are made. Cognition and behavior may but must not run in synchrony with seizures, MRI pathology, or immune parameters. Cognitive and behavioral problems are integral aspects of LE and represent important biomarkers of disease acuity, progress, and therapy response beyond and in addition to parameters of immunology, neurological symptoms, and brain imaging. Thus, evidence-based neuropsychological assessments are essential for the diagnostic workup of patients with suspected or diagnosed limbic encephalitis, for treatment decisions, and disease and treatment monitoring.

## 1. Introduction

Cognitive and behavioral changes are frequent features of limbic encephalitis (LE). Therefore, neuropsychological evaluations are an essential element of the diagnostic workup of patients with diagnosed or suspected LE. Moreover, cognition and behavior are relevant outcome parameters regarding the course of the disease and the evaluation and guidance of medical interventions. In clinical practice and for individual patient care, the question arises as to which cognitive and behavioral domains are affected by LE and at what times cognition and behavior should be assessed and which measures are suited.

## 2. Background

LE is an autoimmune disease of the central nervous system with auto-antibodies directed against surface or intracellular antigens of the brain. Auto-antibodies against surface antigens (e.g., leucine-rich glioma inactivated protein 1 (LGI1) and contactin-associated protein-like 2 (CASPR2)) may directly affect the targeted antigen by blocking its function with or without subsequent alterations of synaptic density or by interfering with synaptic protein-protein interactions [1]. In contrast, there is no evidence that auto-antibodies against intracellular antigens (e.g., glutamate decarboxylase (GAD65) and onconeural antigens in paraneoplastic LE) are directly pathogenic. Instead, inflammatory processes involving cytotoxic T-cells are discussed [1].

LE is associated with inflammatory changes primarily affecting mesiotemporal structures of the limbic system. The major morphological and metabolic changes pertain to the amygdala and the hippocampus of the affected hemisphere(s) [2]. On the one hand, the inflammatory process can lead to functional disturbances of the affected structures which may lead to epileptic seizures as well as cognitive and behavioral problems. On the other hand, uncontrolled inflammatory and underlying autoimmune processes may also cause irreversible structural damage and chronic epilepsy. Cell death may be induced by cytotoxic T-cells or by the altered cell signaling even without inflammatory cell infiltration [3]. Accordingly, cognitive and behavioral alterations can be dynamic and reversible or chronic and irreversible. The type and extent of the impairments depend on several etiological factors including (1) the underlying autoimmune process (type of auto-antibodies [4], B- and T-cell activity [5]) (2) the inflammatory-driven functional and/or structural changes [2,6,7], (3) if applicable, epileptic seizures and interictal epileptic discharges [8], (4) immunomodulatory or anti-epileptic treatment effects [9,10,11]. Finally, (5) pre-existent psychiatric comorbidities or (6) psychiatric symptoms caused by LE may also have a negative impact on cognitive performance.

Regarding the functional relevance of the primarily affected mesiotemporal structures, the amygdala is part of the basolateral-limbic circuit that processes emotional valence. The hippocampus is an element of the Papez circuit and of major relevance for declarative long-term memory [12]. The hippocampus is a so-called bottleneck structure and represents the gate to episodic long-term memory. Consequently, LE and the accompanied affection of the limbic system often lead to altered affective states and to an impairment of episodic long-term memory.

## 3. Which Neuropsychological Functions Are Affected?

In a position paper, Graus et al. [13] present a clinical approach to the diagnosis of autoimmune encephalitis. Regarding neuropsychological problems in limbic encephalitis, they point out subacute “working memory deficits” or “short-term memory loss”, respectively. However, the position paper does not provide any evidence for this assertion and only a few of the available publications report working memory deficits, but when, these are mostly present in combination with the to-be-expected episodic long-term memory deficits. That is why we assume a terminological blunder (short- vs. long-term memory) which calls for a correction (see also [14,15]). Short-term and working memory functions are defined as the maintaining or processing of information in a time range of seconds to a few minutes and they are mediated by a frontoparietal network [16] and thus independent from the limbic system. Tragic lessons from early epilepsy surgical cases (cf. H. M.) disclosed a clear dissociation between short- and long-term memory [17]. However, immediate recall starts to become dependent on the hippocampal memory system when the memory load exceeds the limited short-term memory capacity (memory span) [18]. 

In the respective guidelines of the German Neurological Society (DGN) that were first published in 2012, episodic memory deficits as well as disturbed affect with emotional lability (“affect incontinence”) are correctly listed as two of three possible clinical symptoms. Consequently, neuropsychological and behavioral examinations are an essential part of the diagnostic workup of patients with suspected limbic encephalitis. However, according to the DGN guidelines, the presence of a corresponding mnestic deficit or an affective abnormality is neither necessary nor sufficient for the diagnosis, since temporal lobe seizures would also be sufficient although not mandatory for a clinical “limbic” syndrome. 

An excellent overview of cognitive impairment in the acute and post-acute phases of different auto-antibody defined subtypes of LE is provided by Gibson et al. [4]. The overview indicates that confusion can be another relevant symptom in the acute phase in all subtypes of LE but predominantly in LGI1 and gamma-aminobutyric acidB-receptor (GABAbR) associated LE.

### 3.1. Episodic Long-Term Memory

In the majority of cases with LE, an impairment of the hippocampus-dependent episodic memory formation can be demonstrated [14]. The severity of the mnestic deficit may depend on the specific pathological auto-antibodies: Patients with auto-antibodies against elements of the potassium channel complex (VGKC) are significantly more affected than patients with auto-antibodies that are directed against intracellular GAD65 [9]. Although the former group present with a significant impairment, the latter often show a milder borderline deficit [9]. Meanwhile the VGKC subgroup is further stratified for specific antigens of the VGKC, i.e., CASPR2 and LGI1 [19]. A recent overview of neuropsychological studies regarding LGI1-positive LE is provided by Griffith et al. [20].

Potential etiological factors include a possibly reversible inflammation-related hippocampal dysfunction, structural hippocampal damage (i.e., significant cell loss) and any interictal epileptic discharges. In a recent study we demonstrated a link between T- and B-cell activity (as assessed by flow cytometry) and neuropsychological performance in patients with LE [5]. If the dynamic factors can be controlled at an early stage through successful immunomodulatory and, if necessary, anti-epileptic treatment, a cognitive recovery would be anticipated [9]. On the other hand, in case of structural hippocampal damage (i.e., hippocampal sclerosis), a persistent mnestic deficit can be expected [19,21,22].

### 3.2. Accelerated Long-Term Forgetting

In contrast to the classic episodic memory deficits that occur after mesiotemporal damage, some case reports [23,24] of patients with LE indicate a different kind of mnestic phenomenon termed accelerated long-term forgetting (ALF). Affected patients report that they can initially remember complete episodes and newly acquired content for a limited period of time from hours to several days, but then disproportionally forget these memory contents with longer time intervals. Since this phenomenon can also occur in temporal lobe epilepsies without evidence of an obvious autoimmunological etiology, e.g., in patients with transient epileptic amnesia and sometimes even in genetic epilepsies [25], ALF does not appear to be specific to LE. Nevertheless ALF should be considered in clinical practice, especially when subjective memory complaints cannot be confirmed by standard memory tests with limited retention intervals [26]. In a systematic investigation of ALF in patients with diagnosed or suspected limbic encephalitis three definitions of ALF were concomitantly differentiated and results indicated ALF in up to two-third of the analyzed sample [27]. The authors of that study discuss a non-lateralized fronto-limbic dysfunction in LE instead of a lateralized hippocampal functional disturbance. 

### 3.3. Retrograde Memory Deficits

As a third variant of memory dysfunctions, LE may go along with retrograde memory deficits that primarily affect the autobiographical-episodic memory and rarely also the memory for public events [28,29,30]. The degree of the memory loss is very variable ranging from months to many years. A more specific type of retrograde memory deficits may be circumscribed memory loss of specific events of usually high emotional relevance, e.g., a wedding or funeral. Sometimes there are still vague memories of the events but with a noticeable loss of both emotional valence and autonoetic awareness [24]. This constellation appears compatible with an inflammation-related dysfunction of the amygdala [31]. In contrast to anterograde memory impairments, no systematic dependence of retrograde memory deficits on specific auto-antibodies or on the lateralization of pathology has been demonstrated to date. The retrograde semantic memory deficits appear partially reversible after successful immunomodulatory therapy [29,30]. This, however, does not necessarily mean that lost personal memories are regained. 

### 3.4. Deficits in Attention and Executive Functions

Besides memory impairment, LE may also affect attention and executive functions [9,32] which are primarily subserved by extratemporal (mostly frontal) brain networks and immunomodulatory therapy can lead to a respective recovery [9,33]. These findings would be compatible with the above assumption of a fronto-limbic dysfunction in LE. In this regard other potentially relevant impact factors need to be taken into consideration such as propagation phenomena of interictal epileptic discharges from the limbic system into the frontal lobes [34] or adverse side effects of anti-epileptic pharmacotherapy [11].

### 3.5. Psychiatric Symptoms

Besides the disturbed affect in terms of hyper- or hypoemotionality further behavioral changes and psychiatric symptoms have been described in patients with LE including depressive states, anxiety disorders and panic attacks, irritability and psychotic elements [24,35,36]. A recent study from the United Kingdom [37] claims that pathologic tearfulness is a common (50% of the examined patient sample) post-acute symptom in LE that has been newly described by the authors. To what extent this symptom can actually be differentiated from the "affect incontinence" listed in the DGN guideline mentioned above remains questionable. 

## 4. Which Neuropsychological Measures Are Suited?

The most decisive criterion for test selection is available evidence that the measure is valid and suited for the matter of investigation [38]. In case of limbic encephalitis, the neuropsychological measures need to be sensitive to (mesio)temporal pathologies and dysfunctions. Further test selection criteria besides objectivity, reliability, and validity include the quality and range of normative data (sample size, representativity, stratification for age etc.), the suitability for follow-up assessments (availability and number of parallel versions, information about practice effects, test-retest norms) as well as the time needed for applying and scoring the tests. Ideally, the whole evidence-based test battery would be standardized based on one normative sample. This would allow for an evaluation of significant intraindividual discrepancies within the average to above average range (for example, a statistically significant inferiority of verbal compared to visual-spatial memory performance in an average neuropsychological profile could already indicate a dysfunction of the language dominant (mostly left) temporal lobe and vice versa.). Given their non-specificity, dementia screenings (e.g., *Mini–Mental State Examination* (*MMSE*)*, *Montreal-Cognitive-Assessment** (*MoCA*) etc.) and intelligence test batteries are not suited to assess (mesio)temporal dysfunctions [38]. 

Given the long-standing experience gained in the context of epilepsy surgery, neuropsychological measures are known that are sensitive to (mesio)temporal pathologies and dysfunctions as well as to (mesio)temporal resections. As it stands, word list learning tests such as the *Rey Auditory Verbal Learning and Memory Test* (*RAVLT*) and its derivates [39], are particularly suited for the valid assessment of functions mediated by the temporal structures of the language dominant (mostly left) hemisphere [9,40]. They have frequently been employed in cognitive studies in patients with limbic encephalitis [14]. The RAVLT assesses episodic long-term memory. The free recall of learned items after a delay of 20–30 min represents the most sensitive parameter for mesiotemporal dysfunctions [18]. Parameters of learning, in contrast, are more closely connected to neocortical temporal and frontal lobe [40]. Functions of the temporal lobe within the non-dominant (usually right) hemisphere can be assessed via different nonverbal visual-spatial learning and memory tasks. The most frequently employed tests in patients with LE are the revised version of the *Diagnosticum für Cerebralschädigung* (*DCS-R* [41]) and the *Rey-Osterrieth Complex Figure Test* (*ROCF* [42]) [14]. Although the latter is not suited for reassessments, the DCS-R proved itself in practice with regard to the monitoring of patients with limbic encephalitis [14]. Studies indicate that DCS-R learning performance is the most sensitive parameter for right temporal lobe dysfunction [9,41]. An alternative could be the *Brown Location Test* (*BLT* [43]) which is also sensitive to right temporal lobe dysfunction [44,45], but studies in LE have not yet been published.

The evaluation of autobiographical memory deficits is challenging given that each biography is unique. The most pragmatic approach in clinical practice would be an emphasis on a careful semi-structured anamnesis, at best, in combination with an external anamnesis by close relatives. This approach may be flanked by available standardized procedures such as the *Autobiographical Memory Interview* (*AMI*) [46] which, however, may easily miss the often circumscribed loss of recent biographic memories outlined above. 

The assessment of ALF is also challenging since there are no established approaches. Modifying existing learning and memory tests by adding an extended retention interval would be a pragmatic approach [26]. At our department the verbal learning and memory test was extended by an additional free recall and recognition trial after 1 week [27]. However, sufficient normative data need to be gathered first and, given that this long-term assessment needs to be unannounced (otherwise patients could reiterate or write down the list of words), the approach is only suited for status diagnostics and unfortunately not for subsequent follow-up assessments. 

The examination of attention and executive functions should include psychomotor speed, cognitive flexibility, response inhibition, phonemic fluency and working memory. For example, the EpiTrack^®^ [47] assesses and integrates these aspects and was devised for follow-up evaluations allowing for a cognitive monitoring of anti-epileptic pharmacotherapies [11] and it is also employed for cognitive follow-up examinations in LE [9]. The name of the test implicates closeness to epilepsy, but in fact it is a disease-independent test of executive function in general.

Eventual behavioral changes and affective disorders in suspected or confirmed LE should be systematically taken into account. However, an objective assessment is not possible here. Consequently, diagnostics are based (1) on the self-report and (2), if possible, on external anamnesis by relevant relatives (3) in addition to the application of behavioral and depression inventories. Diagnostics should at least cover depressive symptoms and the aforementioned alterations of emotionality considering both directions (excessive vs. lack of emotional reactions). The *Beck Depression Inventory* (*BDI*) has proven useful for the assessment of depressive symptoms. Relying just on depression, however, may neglect behavioral changes in other domains. Up to now, no procedure for behavioral assessment has been established so far in LE. At our department we employ a behavioral inventory named *Fragebogen zur Persönlichkeit bei zerebralen Erkrankungen* (*FPZ*; in English: *Clinical Personality Scales*; *CPS*), which has been designed for brain damaged patients, and which, in addition to many other potentially relevant behavioral aspects, also covers hyper- and hypoemotionality [48]. 

Table 1 provides an overview of the discussed cognitive and behavioral problems in limbic encephalitis and the approaches to assess them.

## 5. When Should Neuropsychological Evaluations Be Conducted?

Neuropsychological evaluations should ideally be carried out at an early stage of the disease process, at best before initiation of immunomodulatory or, if required, anti-epileptic therapy. This initial assessment serves as baseline for the valid evaluation of subsequent changes in the course of the disease and after treatment cycles. Particularly in patients who initially show mild or no impairment, in those who are auto-antibody negative, or those who do not show characteristic MRI changes, a follow-up evaluation and proof of a stable, recovering or progressively deteriorating symptomatology can provide valuable hints for treatment or treatment escalation. If a standardized neuropsychological test battery cannot initially be carried out due to a delirious state of the patient, regular qualitative bedside tests [49,50] or simple or more complex behavioral rating systems (Modified Rankin Scale [51] or Scores of Independence for Neurologic and Geriatric Rehabilitation (SINGER) [52], respectively) should be considered in order to document the cognitive recovery at this early stage of the disease. 

Neuropsychological follow-up assessments are always indicated when (1) a significant subjective deterioration or improvement in cognition in the course of the disease needs to be verified or (2) the necessity for a change or escalation of the immunomodulatory or anti-epileptic therapy is assessed or (3) the success of a treatment or a treatment cycle should be evaluated. In this regard, cognition, affect, and behavior are essential outcome parameters besides and beyond structural brain imaging, auto-antibody diagnostics and seizures. 

Structured and coordinated multimodal follow-ups are recommended with a parallel assessment of all relevant outcome parameters. It is very important to know that changes in the different outcome parameters may occur in a coherent manner (e.g., a decline of auto-antibody load is associated with a regressive mesiotemporal swelling, a recovery of memory functions and seizure control), but changes may as well appear quite independent of each other or may follow different time courses [53]. 

A follow-up investigation after a completed treatment or treatment cycle should be scheduled in a representative time window in which a therapeutic effect can be anticipated and no flare-up of the inflammatory process is to be expected yet. Neuropsychological assessments during an ongoing cortisone pulse therapy should be avoided due to the potential influence on memory [10]. 

In general, the cognitive monitoring needs to be strategic and targeted given that the number of reassessments is limited by the number of available parallel forms of the employed tests. 

There is, however, a rule when the same tests are applied repeatedly. Performance should not become worse and, in the expectation of practice effects, even a stable performance might indicate a negative outcome [38].

## 6. Exemplary Cases Demonstrating the Cognitive Monitoring of LE

**Figure 1:**
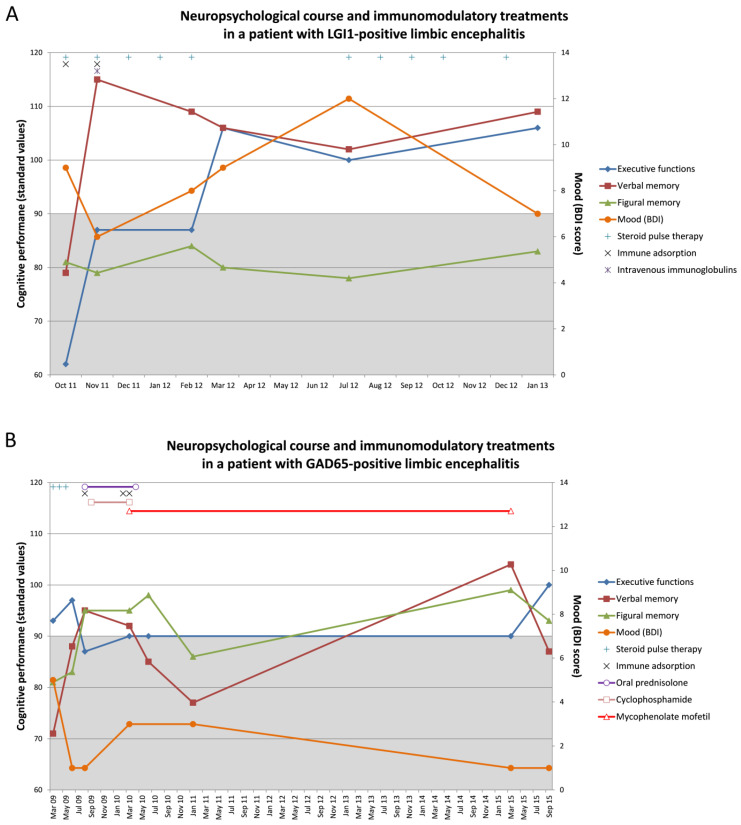
Exemplary cases demonstrating the cognitive monitoring of patients with limbic encephalitis (LE). The figures show the neuropsychological course and immunomodulatory treatments in (**A**) a patient with LGI1-positive LE and (**B**) a patient with GAD65-positive LE. The left *y*-axis refers to the cognitive performances which are presented in standard values. The below average range (i.e., a standard values below 90) is highlighted in grey. The right *y*-axis refers to the Beck Depression Inventory (BDI) score. A BDI score >10 would indicate depressed mood.

Two case reports with longitudinal neuropsychological assessments may present examples of the necessity to monitor cognition over time to reveal the dynamics during LE and treatment effects (Figure 1). The repeated measurements demonstrate in part considerable dynamics in performance over time. A single assessment may easily lead to wrong conclusions. Therefore, dynamics in cognition should be considered to be an additional essential clinical feature of an active LE. Cognitive decline, independent of seizures, may be a signal for initiating, escalating or changing treatment. The need for follow-up assessment to evaluate and justify the actions taken is self-evident. 

The first patient (71 years, female) was admitted to the clinic in September 2011 with new-onset seizures (with very frequent and typical facio-brachial dystonic seizures, daily focal impaired awareness seizures, and one past single focal to bilateral tonic-clonic seizure), as well as confusion and memory deficits regarding recent events (e.g., the death of a near friend). An initial dementia screening (MMSE: 22/30) confirmed deficits in orientation and episodic memory that would be compatible with dementia. MRI revealed a right frontal meningioma, and positron emission tomography/computed tomography (PET/CT) disclosed a bilateral frontotemporal hypometabolism that would be compatible with frontotemporal dementia. However, she was finally diagnosed with a non-paraneoplastic LE with LGI-1 auto-antibodies in serum (immunofluorescence test: 1:1000) and CSF (1:10). Whole-body PET/CT revealed no malignancies. The first comprehensive neuropsychological assessment in October 2011 indicated episodic memory deficits for verbal and figural information and a severe executive dysfunction (Figure 1A). The profile was interpreted as representing a frontotemporal dysfunction with bilateral affection of the temporal lobes. According to the clinical inventories mood and affect were not impaired, but another questionnaire showed an impaired drive and increased impulsivity (cave: levetiracetam monotherapy [54]). The patient received steroid pulse therapy (Figure 1A) in addition to anti-seizure medication. Immunotherapy was escalated by 14 subsequent cycles of immune adsorption, and she finally received intravenous immunoglobulins. This led to a significant clinical improvement and seizure freedom in November 2011. At that time, the patient was auto-antibody negative. Going along with this, verbal memory (primarily mediated by the left hemisphere) improved significantly by more than three standard deviations while figural memory (primarily mediated by the right hemisphere) remained to be poor (Figure 1A). Mood varied in a borderline zone and showed signs of improvement as well at that time. Executive functions improved with a delay in March 2012. The MRI now showed a discrete volume difference regarding the amygdala (pathology right > left). In July 2012, the patient experienced a seizure relapse with a new somatosensory semiology. Before, between November 2011 and July 2012, verbal memory mildly declined but remained within the normal range. In parallel mood became steadily worse. Figural memory continued to be stable and poor. The patient was treated again with steroid pulse therapy for another 6 months, became seizure free early on, and performances appeared to have stabilized when reexamined in January 2013 (Figure 1A). Anti-seizure-medication was kept stable with levetiracetam monotherapy and until July 2012. Thereafter lamotrigine was given add on. Overall, the neuropsychological data indicate a covariation particularly of executive functions and verbal memory with the course of the disease and its treatment, whereas the profound figural memory impairment was largely unmodulated. Reasons for this can be seen in the possibility of an already permanent right temporal damage or compensatory processes in terms of a scarification of right hemispheric in favor of left hemispheric functions [55,56]. 

The second patient (49 years, male) suffered from focal impaired awareness seizures with a temporal semiology since age 30. The EEG showed right temporal epileptic discharges and MRI indicated mild right mesiotemporal hyperintensities of the amygdala and the hippocampus. PET findings were concordant with a right mesiotemporal hypometabolism. Whole-body PET-CT revealed no malignancies. The first neuropsychological assessment in March 2009 disclosed episodic memory deficits pronounced for verbal information (deficit left temporal > right temporal; Figure 1B). A language-fMRI, however, revealed typical left hemispheric language dominance. The depression inventory was unsuspicious, but the behavioral inventory revealed significant hypoemotionality. The patient had type 1 diabetes mellitus. With GAD-auto-antibody titer in serum (1:n) of up to 32,000, the patient was diagnosed with a non-paraneoplastic GAD65 LE. Apart from anti-seizure-medication, he received steroid pulse therapy from March 2009 to May 2009. In this time, figural and verbal memory in particular improved from a level of 2–3 standard deviations below average to normal early on along with immunomodulatory treatment while executive functions showed a mild decline (Figure 1B). In August 2009, the patient received oral prednisolone (until April 2010) and 13 cycles of immune adsorption resulting in a significant decline of the GAD auto-antibodies titer in serum (1:n) to 2000. In September 2009, cyclophosphamide was given until March 2010. In February and March 2010, the patient received 14 cycles of immune adsorption and he was put on mycophenolate mofetil. Cognitive performances stayed stable until March 2010. Thereafter, verbal memory declined to a poor performance level followed by a delayed deterioration of figural memory performance later in January 2011. Afterwards, the patient was followed-up for every six months without cognitive assessments until March 2015. In that time interval, the patient still suffered from clusters of seizures, but the clinical condition and subjective memory appeared stable. Given the increased susceptibility to infections, treatment with mycophenolate mofetil was discontinued in March 2015 followed, however, by a decline of verbal memory in particular. There were no more immunomodulatory treatment attempts. At the final visit in September 2015, the patient still suffered from right temporal lobe seizures. However, the neuropsychological assessment still indicated a verbal memory deficit (Figure 1B). Anti-seizure medication was a combination of levetiracetam and lamotrigine until June 2010, and thereafter a combination of levetiracetam and primidone. In this patient the memory impairments only partially fitted to the MRI abnormalities and the epileptic focus. However, both memory functions covaried with the course of the disease and the extensive treatment attempts. Compared to this executive functions and mood appeared less responsive. As it stands, most GAD65-positive LE take a chronic disease course due to a poor therapy response [57]. Only two cases have yet been described with a complete recovery after very early immunomodulatory treatment [57].

An up-to-date overview of current treatment strategies in the management of autoimmune encephalitis is provided by Bien [57]. 

## 7. Conclusions

Limbic encephalitis (LE) can negatively affect cognition, mood and behavior. On the cognitive level, LE is primarily associated with different variants of mostly subacute episodic long-term memory dysfunction but also with impairments in attention and executive functions. On the behavioral level, patients with LE often show altered affective states, but other and partially severe psychiatric symptoms have been described as well. Cognition, affect and behavior can recover after immunomodulatory treatment as long as no persistent structural damage has been induced. 

An evidence-based neuropsychological baseline assessment for supporting the diagnosis of LE should ideally be conducted before treatment initiation. Repeated assessments for demonstrating disease- or treatment-related disease dynamics should become an essential part of the diagnostic workup of patients with evident or suspected limbic encephalitis. Therefore, neuropsychology contributes to the diagnosis of LE, it is an important outcome parameter for monitoring the course of the disease and the success of therapeutic interventions, and therewith may guide treatment decisions.

## Figures and Tables

**Table 1:** Overview of cognitive and behavioral problems in limbic encephalitis and approaches to assess them.

Affected Neuropsychological Functions	Major Deficits	Assessment
Anterograde episodic long-term memory deficits	Reduced learning capacity and/or impaired delayed free recall performance. Concerns verbal learning and memory but often also and sometimes only figural/visual-spatial memory; different from unilateral hippocampal sclerosis not necessarily lateralized (left/right verbal/nonverbal)	A combination of verbal and nonverbal memory tests with proven sensitivity to left and right mesiotemporal lobe pathologies
Accelerated long-term forgetting	Mostly unimpaired memory performance with standard retention intervals (up to 1 h) with subsequent disproportional loss with longer retention intervals	Adding extended retention intervals (e.g., 1 week after learning) to existing learning and memory tests
Retrograde episodic long-term memory deficits including loss of recent circumscribed autobiographic episodes	Insular loss of biographic episodic content, semantic content may be preserved or relearned, often loss of the whole event and not of individual aspects, loss of emotional attachment and autonoetic awareness; loss of visual imagination, reconstruction	A careful self-anamnesis, at best, in combination with an external anamnesis by close relatives
Deficits in attention and executive functions	Non-specific	Tests assessing psychomotor speed, cognitive flexibility, response inhibition, phonemic fluency and working memory
Psychiatric symptoms	Emotional instability, affect incontinence, tearfulness, but also hypoemotionality, and symptoms of anxiety and depression, panic attacks, irritability and psychotic elements	Self-anamnesis and if possible external anamnesis by relevant relatives as well as systematic self-assessments addressing affective disturbances and further psychiatric symptoms
